# Attempt to Develop Rat Disseminated Intravascular Coagulation Model Using Yamakagashi (*Rhabdophis tigrinus*) Venom Injection

**DOI:** 10.3390/toxins13020160

**Published:** 2021-02-18

**Authors:** Akihiko Yamamoto, Takashi Ito, Toru Hifumi

**Affiliations:** 1 Management Department of Biosafety and Laboratory Animal, National Institute of Infectious Diseases, Tokyo 208-0011, Japan; 2 Department of Systems Biology in Thromboregulation, Kagoshima University Graduate School of Medical and Dental Sciences, Kagoshima 890-8520, Japan; takashi@m3.kufm.kagoshima-u.ac.jp; 3 Emergency and Critical Care Medicine St. Luke’s International Hospital Tokyo 104-8560, Japan; hifumitoru@gmail.com

**Keywords:** rat disseminated intravascular coagulation model, Yamakagashi (*Rhabdophis tigrinus*) venom, lethality, thrombocytopenia, D-dimer, anti-Yamakagashi equine antibody

## Abstract

Disseminated intravascular coagulation, a severe clinical condition caused by an underlying disease, involves a markedly continuous and widespread activation of coagulation in the circulating blood and the formation of numerous microvascular thrombi. A snakebite, including that of the Yamakagashi (*Rhabdophis tigrinus*), demonstrates this clinical condition. Thus, an animal model using Yamakagashi venom was constructed. Yamakagashi venom was administered to rats, and its lethality and the changes in blood coagulation factors were detected after venom injection. When 300 μg venom was intramuscularly administered to 12-week-old rats, (1) they exhibited hematuria with plasma hemolysis and died within 48 h; (2) Thrombocytopenia in the blood was observed in the rats; (3) irreversible prolongation of prothrombin time in the plasma to the measurement limit occurred; (4) fibrinogen concentration in the plasma irreversibly decreased below the measurement limit; and (5) A transient increase in the plasma concentration of D-dimer was observed. In this model, a fixed amount of *Rhabdophis tigrinus* venom injection resulted in the clinical symptom similar to the human pathology with snakebite. The use of the rat model is very effective in validating the therapeutic effect of human disseminated intravascular coagulation condition due to snakebite.

## 1. Introduction

Disseminated intravascular coagulation (DIC), a severe clinical condition caused by an underlying disease, involves a markedly continuous and widespread activation of coagulation in the circulating blood and the formation of numerous microvascular thrombi. DIC is one of the most severe complications seen in patients suffering from sepsis, cancer, acute leukemia, abruption of the placenta, and trauma. It is difficult to diagnose and treat. Moreover, it is associated with a poor prognosis as it plays a significant role in organ failure and related mortality [1,2,3]. Sepsis is the most common underlying disease causing DIC. Several studies have been conducted on sepsis induced DIC models [4,5].

Meanwhile, DIC or DIC-like syndrome after envenomation by snakes was also reported [6,7,8]. Venoms from snakes have several kinds of proteins and toxic activities [9]. Hematologic abnormalities are the most common effects of snakebite envenoming globally. Venom-induced consumption coagulopathy (VICC) is the most common and most important symptom [10]. Other hematologic abnormalities include anticoagulant coagulopathy and thrombotic microangiopathy. VICC is a venom-induced activation of the clotting pathway by procoagulant venoms, which results in clotting factor consumption and coagulopathy. The type of procoagulant venom differs between snakes and can activate prothrombin, factor X, and factor V or consume fibrinogen (FIB) [11]. 

Yamakagashi (*Rhabdophis tigrinus*), belonging to the family Colubridae, is a rear-fanged venomous snake. It has short fangs (about 2 mm) with no groove. This venomous snake is widespread in East Asia. In Japan, this snake is common in the paddy fields and feeds chiefly on frogs [12]. The severe case of the bite is rare because the venom can be introduced into the skin of the person only when they are attacked with the rearmost fangs. There are reports of several cases of bites requiring treatment every year [13]. As to the chemical characterization of Yamakagashi’s venom, the higher molecular weight fraction of the venom contained a prothrombin activator. Both the prothrombin time (PT) and the activated partial thromboplastin time (APTT) of human plasma were shortened by the addition of this snake venom. The thrombin formation was estimated by the uses of SDS-PAGE and chromogenic substrates. These venom fractions also possessed specific proteinase activity on human fibrinogen, but the substrates for matrix metalloproteinase, such as collagen and laminin, were not hydrolyzed [14]. In an in vivo experiment using mice, not only local hemorrhage but also systemic subcutaneous, pulmonary, and subendocardial hemorrhage were observed, as well as microthrombi in the alveolar capillaries and glomerulus [15]. In cases of bites, systemic hemorrhage, DIC, acute renal failure, etc., have been observed [16,17], and two fatal cases of acute pulmonary edema and cerebral hemorrhage have been reported [18,19]. Currently, an unapproved drug, Yamakagashi antivenom, is used as a treatment method for Yamakagashi envenomation, but there are restrictions on its use. We aimed to create a DIC model using rats to explore the possibility of using approved drugs such as anti-DIC drugs for the treatment of this poisonous snake.

Since the clinical DIC situation can be created by the envenomation as described above, this study aimed to create a DIC model of the envenomation by administering it to rats using Yamakagashi venom. In this model, the intramuscular administration of a fixed amount of *R. tigrinus* venom resulted in blood coagulation changes, and fibrinolytic factors over time resembled the pathology of human blood in envenomation.

## 2. Results

### 2.1. Effect on Lethality Induced by Yamakagashi Venom in Rats

**Figure 1:**
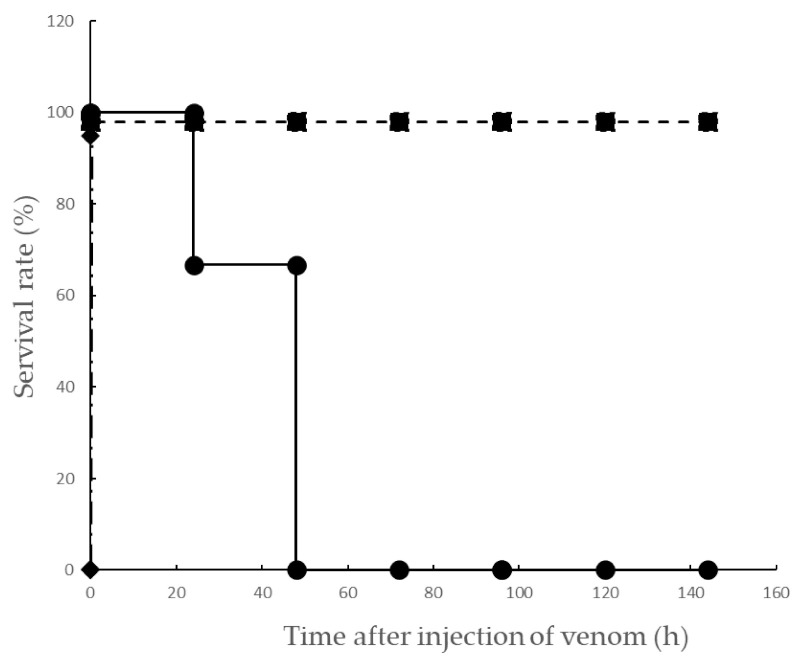
Lethal dose of Yamakagashi venom. Survival time after intravenous (IV) injection of 150 μg venom (◆). Survival time after IM administration of 150, 200, and 250 μg venom (■). Survival time after intramuscular (IM) administration of 300 μg venom (●). Survival time of rt intramuscularly administered 300 μg venom and antivenom 2 h after venom administration (▲). Each group consists of 3 rats.

Based on clinical reports, DIC is clearly caused by Yamakagashi envenomation. Since a report revealed that the pathological changes after the bite of Yamakagashi were analyzed in rats, the minimum dose of Yamakagashi venom that was lethal to rats was investigated. Rats were administered with Yamakagashi venom with different amounts (150, 200, 250, and 300 μg), and their survival time was observed (Figure 1). The administration routes of the venom were: (1) intravenous and (2) intramuscular (IM). The intravenous administration of 150 μg venom to rats resulted in their immediate death. However, when 150, 200, 250, and 300 μg were intramuscularly administered to individual rats, all of them survived up to 144 h after administration of 150, 200, and 250 μg, and all died within 48 h after the administration of 300 μg. When Yamakagashi antivenom was intravenously administered 2 h after IM administration of 300 μg venom, all rats survived until 144 h later (*p* < 0.05, log rank test). The rats that were intramuscularly administered 300 μg Yamakagashi venom exhibited hematuria until death. The plasma collected from these rats demonstrated significant hemolysis until death. Conversely, the plasma collected from rats administered 250 μg venom exhibited significant hemolysis once but recovered 8–24 h following venom administration. Moreover, when a specific antibody was administered after 2 h of the envenomation, the plasma collected from the rat showed significant hemolysis once but recovered 4–8 h after venom administration (Table 1).

### 2.2. Effects of Platelet Counts of Rat Plasma Injected with Yamakagashi Venom

**Figure 2:**
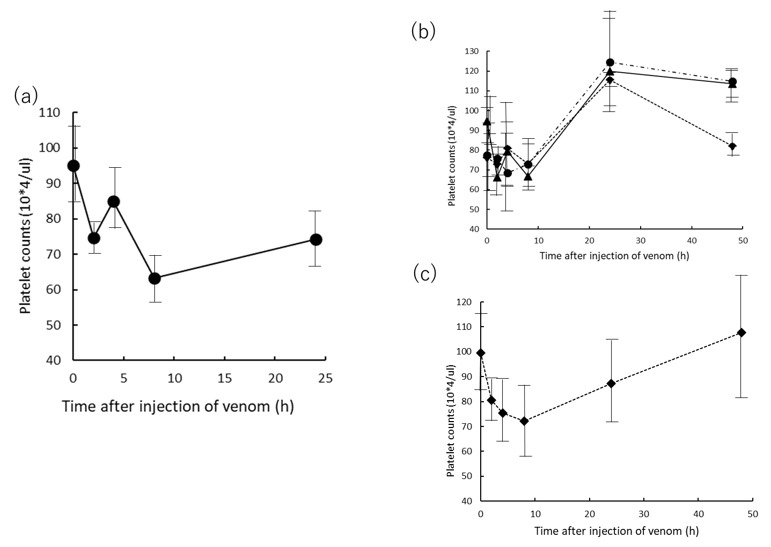
Time course of platelet count after IM administration of Yamakagashi venom. (**a**) 300 μg venom was intramuscularly administered to rats. Closed circle indicates the mean platelet counts. (**b**) Platelet counts of blood from rat intramuscularly administered 150 μg venom (●), 200 μg venom (◆), and 250 μg venom (▲). (**c**) Platelet counts of blood from rat intramuscularly administered 300 μg venom and antivenom 2 h after venom administration (◆). Each group consists of 3 rats. Values were mean ± SD.

To confirm the hemolysis phenomenon of hematuria and collected plasma in rats administered with venom, the number of platelets at each blood collection was measured (Figure 2). Blood was collected from rats administered with 300 μg venom every 0, 2, 4, 8, and 24 h, and the platelet count was measured. Figure 2a presents the mean and standard deviation (SD) of the three independent test results. In the 300 μg venom group, the mean platelet count decreased up to 8 h after venom administration and demonstrated slight recovery to baseline after 24 h. Conversely, in the blood collected from rats to which 150, 200, and 250 μg venom was administered (Figure 2b), the platelet count exhibited the lowest value 8 h after venom administration, but after 24 and 48 h, a recovery in the platelet count was observed. Similarly, in rats treated with the antivenom (Figure 2c), the platelet count decreased once, but recovered to the value before venom administration 24 h later Statistics between the groups of rats receiving 300 μg of venom, the groups of rats receiving 150, 200, and 250 μg of the venom, and the group receiving antitoxin treatment 2 h after administration of 300 ug of toxin, respectively. A comparison was made, and we found that the 300 μg group was significant at a 1% risk rate with each of the other groups. In addition, among the groups other than the 300 ug group, a significant difference was detected between the 150 ug and 200 ug groups with a risk rate of 5%, but there was no significant difference between the other groups.

### 2.3. Effects of Prothrombin Time of Rat Plasma Injected with Yamakagashi Venom

**Figure 3:**
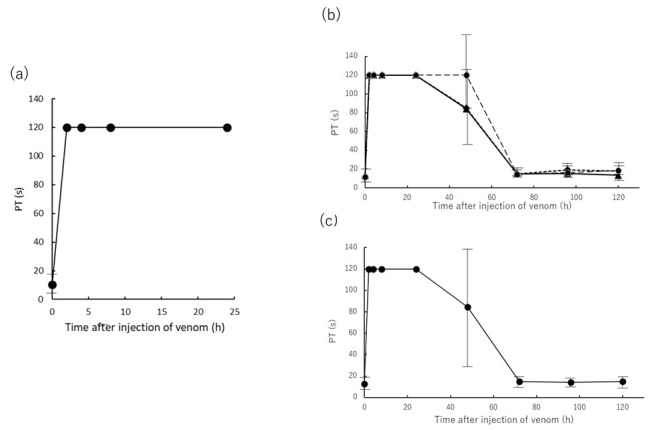
Time course of PT after IM administration of Yamakagashi venom. (**a**) 300 μg venom was intramuscularly administered to rats. Closed circle indicates the PT; (**b**) PT of plasma from rat intramuscularly administered 150 μg venom (●), 200 μg venom (◆), and 250 μg venom (▲); (**c**) PT of plasma from rat intramuscularly administered 300 μg venom and antivenom 2 h after venom administration (●). Each group consists of 3 rats. Values were mean ± SD.

Subsequently, the blood coagulation factor of rats intramuscularly administered Yamakagashi venom was performed. First, prothrombin time (PT) was measured (Figure 3. Plasma PT in venom-treated rats was observed to be prolonged for 2 h after venom administration and did not recover even after 24 h beyond the 120 s measurement limit (Figure 3a–c). Rats treated with 150, 200, and 250 μg of venom and rats treated with 300 μg of venom and then antivenom returned to their original state after 72 h (Figure 3b,c). Statistical analysis of these groups did not detect the presence or absence between any of the groups.

### 2.4. Effects of FIB Concertation of Rat Plasma Injected Yamakagashi Venom

**Figure 4:**
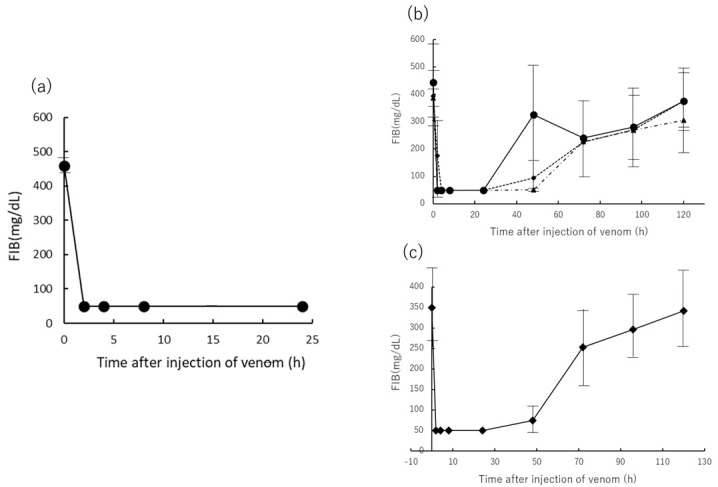
Time course of fibrinogen (FIB) concentration after IM administration of Yamakagashi venom. (**a**) 300 μg venom was intramuscularly administered to rats. Closed circle indicates the mean FIB concentration; (**b**) FIB concentration of plasma from rat intramuscularly administered 150 μg venom (●), 200 μg venom (◆), and 250 μg venom (▲); (**c**) FIB concentration of plasma from rat intramuscularly administered 300 μg venom and antivenom 2 h after venom administration (◆). Each group consists of 3 rats. Values were mean ± SD.

Second, plasma FIB concentration was measured over time as a marker of blood coagulation in venom-administered rats (Figure 4). A decrease in plasma FIB concentration in venom-administered rats was observed from 2 h after venom administration, fell below the measurement limit of 50 mg/dL, and FIB concentration did not recover until 24 h after venom administration (Figure 4a–c). Plasma FIB levels in rats treated with 150, 200, and 250 μg of venom and 2 h after administration of 300 μg of venom recovered by 120 h after venom administration over time (Figure 4b,c). A statistical comparison was made between these groups. The 300 μg group was significant at a 5% risk rate with each of the other groups. In addition, there was no significant difference between the groups other than the 300 μg group.

### 2.5. Effects of D-Dimer Concertation of Rat Plasma Injected with Yamakagashi Venom

**Figure 5:**
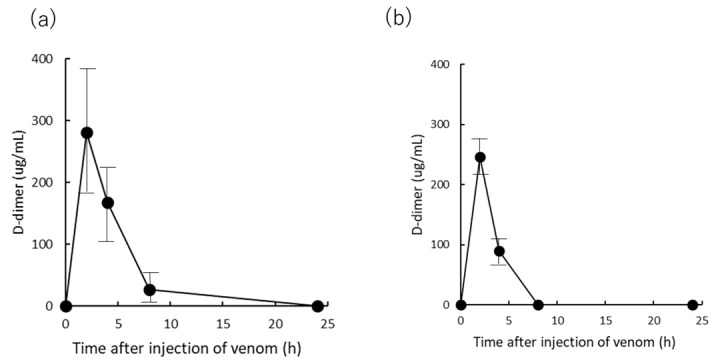
Time course of D-dimer in plasma after IM administration of Yamakagashi venom (**a**) 300 μg venom was intramuscularly administered to rats. Closed circle indicates the mean D-dimer concentration; (**b**) D-dimer concentration of plasma from rat intramuscularly administered 300 μg venom and antivenom 2 h after venom administration (●). Each group consists of 3 rats. Values were mean ± SD.

Finally, the D-dimer concentration was measured as the rats exhibited a sharp decrease in FIB concentration following venom administration, as presented in Figure 4 (Figure 5). The D-dimer concentration in rat plasma after administration of 300 μg venom peaked at 2 h after venom administration, increased to an average of 300 μg/mL, then decreased at 4 and 8 h, and almost disappeared after 24 h (Figure 5a). Furthermore, when antivenom was administered 2 h after 300 μg venom administration, the D-dimer concentration in rat plasma demonstrated the same value 2 h after venom administration but could not be measured 8 h after venom administration (Figure 5b). We compared the changes in the D-dimer values in these two groups and found no statistically significant difference.

## 3. Discussion

This study aimed to construct a DIC rat model using Yamakagashi venom. When 300 μg venom was intramuscularly administered to 12-week-old male Sprague Dawley (SD) rats, (1) the rats demonstrated hematuria with plasma hemolysis and died within 48 h after venom administration; (2) Thrombocytopenia in the blood was observed in these rats; (3) irreversible prolongation of PT in the plasma to the measurement limit occurred; (4) FIB concentration in plasma irreversibly decreased below the measurement limit; and (5) a transient increase in the plasma concentration of D-dimer was observed.

First, regarding lethal activity, it was shown that it is statistically significant that the lethal dose of toxin in rats exceeds 300 μg (Figure 1).

DIC signs in humans are defined as follows: (1) low platelet count, (2) abnormal global clotting assays including PT, (3) low levels of physiological anticoagulant proteases including FIB, and (4) increased FB-degradation products including D-dimer [20]. These signs were like the symptoms of rats in our experiments (Figure 2, Figure 3, Figure 4 and Figure 5).

The decrease in the number of PLTs, the prolongation of PT time, and the decrease in FIB concentration can be inferred to be a secondary decrease in the promotion of coagulation by the administered Yamakagashi venom in the rat, resulting in a deficiency of FIB and PLT [21] (Figure 2, Figure 3 and Figure 4). 

Following this promotion of coagulation, the fibrinolytic system is enhanced, and bleeding tendency occurs (Table 1). This pathway is the normal reaction of blood coagulation in the body [22]. The enhancement of the fibrinolytic system decomposes the produced fibrin to produce fibrin degradation products such as FDP and D-dimer, resulting in a transient increase in D-dimer (Figure 5). In Figure 5, the time-dependent measurement of the amount of D-dimer in the group treated with antivenom 2 h after administration of the same amount of venom as 300 μg IM was almost the same. Antivenom treatment does not appear to be effective in suppressing the transient rise in D-dimer. The reason for this is that the action of Yamakagashi venom on the blood coagulation system is so rapid [21] that even if the venom is neutralized by antivenom treatment, a large amount of fibrin and platelet coagulation clumps are produced within 2 h. It is considered that the subsequent physiological thrombolytic action does not suppress the production of D-dimer. Bleeding tendency occurs due to the rapid decrease in the amount of PLT and FIB consumed due to the promotion of coagulation, and the thrombolytic action caused by the subsequent rapid physiological reaction. This phenomenon is defined as DIC [3].

Yamakagashi venom contains a prothrombin activator, metalloprotease, and cysteine-rich secretory protein, including proteinase that act specifically on fibrinogen [21]. From the results of this experiment, this venom causes a transient decrease in PLT number, prolongation of PT time, and decrease in FIB concentration in rats when administered intramuscularly to SD rats of 150 μg or more but is irreversible at doses of 300 μg or more. It is presumed that this exceeds the recovery function of the blood coagulation system of the living body described above that the amount of toxin administered is above a certain level.

DIC is a systemic pathophysiologic process and not a single disease entity, which results from an overwhelming activation of coagulation that consumes platelets and coagulation factors and causes microvascular fibrin thrombi, which can result in multiple organ dysfunction syndrome from tissue ischemia. Some conditions associated with acute DIC include septic shock, exsanguinating trauma, burns, or acute promyelocytic leukemia [20]. The massive tissue factor stimulus results in excess intravascular thrombin, which overwhelms the anticoagulant systems and leads to thrombosis. Due to the consumption of coagulation factors and platelets, DIC also has a hemorrhagic phase. Treatment of patients with DIC bleeding is recommended to supplement the deficient blood component. [20].

Various kinds of animals have been tried as DIC models in experiments, but the model using rats is the most reported. The DIC rat model expresses the symptoms of DIC using endotoxin, trauma, sepsis, bacteria, scorpion venom, viper venom, and other substances [23,24,25].

Snake envenomation symptoms vary depending on the type of venom possessed by the venomous snake, but some venomous snakes, such as vipers, colubrids, and elapids exhibit DIC symptoms [6,26]. Among them, bites by Yamakagashi, which are widespread in East Asia, are characterized by DIC as the main symptom. In severe cases, the bite by this viper causes intracerebral hemorrhage and death in the patient. A few cases of envenomation were reported in Japan, but until the development of specific antivenoms, about 40% of cases worsened, which led to death [13,27].

This study aimed to construct an animal model that induces DIC symptoms through Yamakagashi venom administration, which induces the main symptoms of Yamakagashi bites, to rats. Intravenous administration of Yamakagashi venom to rats resulted in their immediate death. The experiments conducted by Sakai et al. in pursuit of the pathology of Yamakagashi bites have demonstrated that the severity and timing of symptoms appearing in experimental animals differ depending on the venom administration route [15,18]. In this experiment, IM venom administration was able to induce the symptoms characteristic of DIC, including thrombocytopenia, PT prolongation, decrease in FIB concentration, and transient increase in the plasma concentration of D-dimer, which were observed depending on the amount of venom administered (Figure 2, Figure 3, Figure 4 and Figure 5). By increasing the dose of the venom to 300 μg, severe DIC situation in which these symptoms irreversibly developed and died within 48 h could be reproduced in rats (Figure 1). 

The DIC rat model created this time has the same symptoms and course of dynamics of coagulation factors in blood to human clinical symptoms; the only difference is that the onset time is earlier in rats than in humans [13]. This point is considered to be due to the fact that the amount of venom injected by viper bite varies in humans, whereas in rats, a fixed amount can be administered from the same route; thus, the symptoms naturally occur earlier. Additionally, the peak appearance of D-dimer is about 10 h for humans and 2 h for rats; thus, the above cause is considered [13]. In the rat model established in the current study, the fibrinogen levels fell below the measurement limit at 2 h after injection of venom and did not recover even after 24 h, and D-dimer levels similarly peaked at 2 h. The PT time is also beyond the measurement limit at 2 h and does not recover even after 24 h. In a human case of Yamakagashi bites reported by Dr. Ichiki [13], fibrinogen levels fell below the measurement limit at 5.5 h after Yamakagashi bites, and even after treatment with antivenom, it remained below measurement limit until 33.5 h. FDP peaked at 5.5 h, and D-dimer peaked at 10.5 h. The PT-INR was prolonged beyond the measurement limit at 5.5 h. Thus, remarkable changes in d-dimer, fibrinogen, and PT in human clinical data are also well observed in the current rat models. Considering the differences in body weight and animal species, the difference of 1/2–3 h in the rat model compared to the human clinical data is acceptable. In the future, various treatments can be examined in the current model assuming real clinical situations. Except for the time of onset of symptoms that are thought to be caused by the abovementioned difference in venom dose, the DIC model in rats can be said to well reproduce the clinical symptoms in people with Yamakagashi bites.

For snake envenomation, specific antibody therapy is the first choice [28]. Currently, in medical practice, the globulin fraction immunized in horses and the F(ab)2 component of its pepsin digestion are the antivenoms produced by pharmaceutical companies worldwide as a remedy for snake venom [29]. However, horse antibody therapy sometimes induces severe side effects, such as serum diseases and anaphylactic shock [30]. To avoid these adverse effects, other therapeutic methods need to be tested. Several approved drugs are available for the treatment of DIC [31]. From these, a drug that does not cause side effects, such as horse antivenom, and is effective for Yamakagashi bites should be developed. Furthermore, the development of specific antivenom preparations as approved drugs is such because the application of therapeutic agents such as antivenom preparations for envenomation caused by colubrid is small. From this point as well, it is important to explore the possibility of treatment with other approved antidotes. As an approved antidote for such snake venom, drugs with various specificities called low molecular weight substances have been tried. These drugs have few side effects such as antivenom, reduce the side effects of antitoxin, are specific inhibitors of the analyzed snake venom component, and can be administered in various routes, and are available in the medical field. Easy to use. Examples of such cases have been reported include Varespradib, a specific inhibitor of Phospholipase A2, Flavonoid Myricetin for Metalloprotease, and Nafamostat for Serine protease [32,33,34,35]. Our rat DIC model may be a useful tool to research other therapeutic methods.

Naturally, there are several limitations in the use of the rat DIC model that we have constructed. The male SD rats used in the experiment were 12 weeks old and are difficult to purchase from regular breeders. These rats were used because we needed as large a rat as possible to attach the catheter to. This strain was chosen because SD rats are larger than the other strains, such as Wister strain. It is hard to set polyethylene catheter into the femoral artery and vein for the administration using other rat strain. Whether this DIC model can be reproduced using the most common rat strains, such as Wistar rats, is not clear. The number of rats in each group used in the experiments conducted in this paper was three. The number of animals in one group of 3 is the minimum number from which variance and standard deviation can be obtained. Due to the statistical processing of the experimental results obtained under these conditions, differences in the amount of toxin administered did not result in statistically significant differences in the measured values for some data. There is a limit to the conclusions of this paper because of the inability to perform sufficient statistical processing.

## 4. Conclusions

In this model, the IM administration of a fixed amount of *R. tigrinus* venom resulted in blood coagulation changes, and fibrinolytic factors over time resembled the pathology of human blood in snakebite. The use of our rat DIC model is considered to be very effective in confirming the therapeutic effect of human DIC condition due to snakebite.

## 5. Materials and Methods 

### 5.1. Animal Preparation

Twelve-week-old male Sprague Dawley rats (JAPAN SLC, Inc., Shizuoka, Japan) were housed in separate cages in a temperature-controlled room under a 12 h/12 h light/dark cycle. They were fed a standard laboratory diet and water ad libitum. All surgical and experimental procedures were approved by the Animal Care and Use Committee and conformed to the Guidelines for Animal Experimentation (approval number: 118065, approval date: 13 December 2018.).

Under general anesthesia using 4% isoflurane induction, a polyethylene catheter (PE-60) was inserted into the femoral artery for bleeding. Another catheter (PE-50) was inserted into the femoral vein for the administration of saline solution and drugs. All catheters were filled with heparinized saline (100 U/mL) and placed under the skin and were then opened under the neck to prevent the catheter from coming off when the animal awakened. Blood sampling and drug administration to rats were performed after anesthesia with 4% isoflurane was inducted.

### 5.2. Snake Venoms

Yamakagashi (*R. tigrinus*) venom was extracted from Duvernoy’s gland. Toxic glands were collected from 100 male and female *Rhabdophis tigrinus* with a body length of 80 cm or more throughout Japan. The glands were excised, cut into small pieces, and centrifuged with distilled water, and the supernatant was lyophilized [36]. The venom powder was dissolved in distilled water and was centrifuged again to remove the mucous substance, which contained during venom extraction. The lyophilized venom was stored in a refrigerator. The intravenous LD50 value of the venom was 5.3 μg/20 g mouse [18]. 

### 5.3. Anti-Snake Venom Equine Antibody

*R. tigrinus* antivenom is a freeze-dried product of equine immunoglobulin. It is antivenom against the *R. tigrinus* venom. This immunoglobulin is an F(ab)2 fragment removed from the Fc fragments via pepsin digestion [37].

### 5.4. Experimental Protocols

The rats were initially randomly divided into four groups to evaluate lethality as follows: (a) intravenous injection group (n 3); (b) 150, 200, and 250 μg Yamakagashi venom were administered (each dose: n 3 respectively); (c) 300 μg Yamakagashi venom was administered (n 3); and (d) 0.5 mL Yamakagashi antivenom administered 2 h after Yamakagashi venom (300 μg) administration (n 3). Blood samples were collected at 0, 2, 4, 8, 24, 48, 72, 96, and 120 h after the start of the experiment (500 μL of blood was collected at each time point). An equal volume of saline was administered via the femoral vain after bleeding. Blood samples were anticoagulated with sodium citrate and centrifuged immediately for 10 min at 3000 g, and the plasma supernatant was separated.

### 5.5. Principle of Test in the Measurement of Platelet Counts

Platelet counts were performed using automated system on the ADVIA 2120i (Siemens Diagnostic Solutions, Milan, Italy). ADVIA counts platelets via flow cytometry based on the principles of light scattering. Platelets are identified by their size (<30 FL, low-angle light scatter) and refractive index (*n* = 1.35 to *n* = 1.40 or high-angle light scatter). 

### 5.6. Principle of Test in the Measurement of PT

A sample of the individual plasma was placed into a measuring test tube of CA-50 AutoAnalyzer (Sysmex Co., Kobe, Japan). The coagulation reaction detection method irradiates red light (660 nm) onto a mixture of blood plasma and reagent, detects the change in turbidity (when fibrin clots are formed) as the change in scattered light, and measures the coagulation time (s). It detect within the maximum detection time and measures the result. The typical maximum detection time is 120 s for PT. The coefficient of variation (CV) of the PT measurement is 2% or less. The data of CV are variation coefficients for coagulation times of change in activity (%) obtained from 10 analyses of Dade Behring Ci-Trol Level 1 (control plasma), with PT reagent.

### 5.7. Principle of Test in the Measurement of FIB

A sample of the individual plasma is one tenth dilution with specific reagents and was placed into a measuring test tube of CA-50 AutoAnalyzer (Sysmex Co., Kobe, Japan). The coagulation reaction detection method irradiates red light (660 nm) onto a mixture of blood plasma and reagent, detects the change in turbidity (when fibrin clots are formed) as the change in scattered light, and measures the coagulation time (s). The range of analysis of FIB concentration can be from 50 to 450 mg/dL. The CV of FIB measurement is 4% or less. The data of CV are variation coefficients for coagulation times of change in activity (%) taken from 10 analysis of Dade Behring Ci-Trol Level 1 (control plasma), with Dade Behring Fibrinogen Determination Reagents.

### 5.8. Principle of Test in the Measurement of D-Dimer

The levels of individual plasma D-dimer were measured using a latex photometric immunoassay (LPIA)-NV7 with a LPIA ACE D-Dimer II Kit (LSI Medience, Tokyo, Japan), as reported previously [37]. Briefly, 4 μL of plasma was dispensed with 144 μL of the R-1 solution into the reaction cuvette. After a 2-m stabilizing period at 37 °C, 48 μL of the R-2 suspension was dispensed, which contained latex particles coated with anti D-dimer antibody JIF-23. The increase in turbidity was evaluated during the 7-m reaction period at 37 °C. The analysis range for D-dimer was 0.5–48 μg/mL, and the samples beyond this range were remeasured with dilution. The within-run CV was less than 10%. The cross-reactivity to rat D-dimer was confirmed in the preliminary test using rat plasma samples treated with calcium ion and different concentrations of tissue plasminogen activator.

### 5.9. Statistical Analysis

In the statistical analysis in this paper, the homogeneity of the variance of the data in each group was first tested by the Bartlett test. As a result, when comparing the data groups whose homogeneity was affirmed, the groups were compared by the two-way analysis method. We also used the Mann–Whitney U test to compare groups for data for which homogeneity was denied. For the survival study, the data were analyzed using Kaplan–Meier analysis. Statistical analyses were performed using JMP version 11 software (SAS Institute, Cary, NC, USA). A two-sided probability value of <0.05 was considered statistically significant for all analyses.

## Figures and Tables

**Table 1:** Hematuria and hemolysis of rat after IM injection of Yamakagashi venom.

Group and Time (h)	Hematuria	Plasma Hemolysis
300 μg IM ^1^		
0	−, −, −	−, −, −
2	++, +, +	+, +, +
4	++, +, +	+, +, +
8	++, +, +	+, +, +
24	+, +	+, +
250 μg IM ^2^		
0	−, −, −	−, −, −
2	+, +, +	+, −, −
4	+, +, ++	+, −, +
8	+, −, +	−, −, −
24	−, −, −	−, −, −
48	−, −, −	−, −, −
300 μg IM+Antitoxin ^3^		
0	±, −, −	−, −, −
2	+, ++, +	+, −, −
4	+, +, +	+, −, +
8	−, −, +	−, +, −
24	−, −, −	−, −, −
48	−, −, −	−, −, −

^1^ Rat group injected with 300 μg Yamakagashi venom, ^2^ 250 μg injection, ^3^ 300 μg and antivenom injection. Each group consists of 3 rats.

## Data Availability

Data available in a publicly accessible repository that does not issue DOIs.
